# Role of miRNA in adult ocular tumorigenesis

**DOI:** 10.3389/fmolb.2025.1459761

**Published:** 2025-05-08

**Authors:** Arianna Romani, Elisabetta Melloni, Giada Lodi, Francesca Bompan, Rebecca Foschi, Enrico Zauli, Elena Pozza, Paola Secchiero, Giorgio Zauli, Maurizio Previati, Rebecca Voltan

**Affiliations:** ^1^ Department of Translational Medicine, University of Ferrara, Ferrara, Italy; ^2^ LTTA Centre, University of Ferrara, Ferrara, Italy; ^3^ Department of Environmental and Prevention Sciences, University of Ferrara, Ferrara, Italy; ^4^ Research Department, King Khaled Eye Specialistic Hospital, Riyadh, Saudi Arabia

**Keywords:** miRNA, ocular tumors, uveal melanoma, eye, retinal cancer, eyelid cancer, nanomedicine

## Abstract

In recent years, cancer research has made huge advances also thanks to the discovery of the role of non-coding RNAs in the control of tumorigenesis, tumor proliferation, migration and metastasis and therefore also in the diagnosis and therapy of tumors. This work aims to review the most recent literature involving the study of miRNAs in ocular tumors affecting adult patients. We will introduce the role of miRNAs in tumorigenesis, and we will focus on summarizing the studies on uveal intraocular melanomas in which a role of microRNAs has been demonstrated. Similarly, we will also cover observations on miRNAs and eyelid cancers, especially sebaceous gland carcinoma, and cancers of the conjunctiva and the retina, excluding retinoblastoma which is typically a pediatric-onset tumor. We will summarize specific miRNAs that could be considered as diagnostic molecules or as therapeutic targets against some ocular cancer diseases, indicating their potentialities and limitations, considering also their administration as nanomedicine for the eye.

## 1 Introduction

### 1.1 miRNA

It is a normal finding to retrieve in cancer cells a certain number of genetic damages, most of which are DNA alterations and in particular, mutations. Most of these mutations increase with the age of the patients and are believed to have no specific relevance in the appearance and progression of the tumor, as they are found also in normal tissues. Instead, a very small number of mutations, referred as “driver mutations”, are believed to be advantageous to cell survival and eventually to cellular growth. Cellular overgrowth can lead to the accumulation of other driver mutations in the same tissue up to the appearance of the primitive tumor (Vogelstein et al., 2013). On the other hand, only a small number of driver somatic mutations, and of the affected downstream cellular pathways, have been firmly detected. In a similar way, primitive and metastatic tumors display very similar genetic profiles, suggesting that integrative epigenetic factors distinct from the mere genetic lesion can be involved in the dramatic transition from primitive to metastatic behavior. These epigenetic factors include different activities and classes of molecules, such as DNA methylation, histone post-translational modifications, and the wide family of non-coding RNA (ncRNA). Epigenetics can regulate the interactions among transcription factors, nucleosomes, and DNA, affecting mRNA transcription and, consequentially, the whole cellular phenotype. This aspect is of particular interest for translational medicine because, while DNA alterations cannot be directly addressed by pharmacotherapy, a vast number of molecules can be used as epigenomic drugs in an anticancer perspective. Epigenetic mechanisms can be potential attractive targets of therapeutical intervention, or also, in some cases, some epigenetic molecules themselves can be used as potential drugs to counteract tumor growth and metastasis (Vogelstein et al., 2013; Vezzani et al., 2022).

**FIGURE 1:**
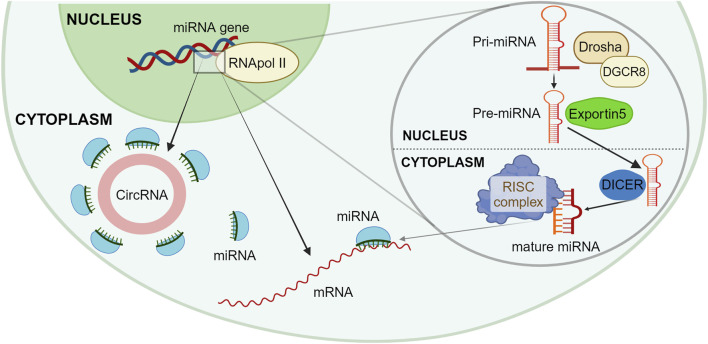
Schematic representation of miRNA synthesis and functions. The RNApol II translates at the nucleus level a new Pri-miRNA, which is then cleaved by the microprocessor complex formed by Drosha and DGCR8 into a Pre-miRNA, with the removal of 5′ and 3′ parts. The Pre-miRNA is moved to the cytoplasm *via* Exportin 5; here, the Dicer complex further cleaves Pre-miRNA originating the mature double strand complex. In the cytoplasm, miRNA can bind to different mRNAs through the RISC complex, thus creating a miRNA-mRNA interaction which slows down mRNA translation and a faster mRNA degradation. Newly created miRNA can get seized by a circular RNA, which acts as a miRNA-sponge, thus not altering the mRNA translation. Created with BioRender.

A post-transcriptional level of epigenetic regulation involves a heterogeneous and not fully explored set of molecules, the ncRNAs. This wide class can act upon both other non-coding or coding RNAs and ultimately regulate most of the biological processes in the human cell (Slack and Chinnaiyan, 2019). Almost 99% of the RNA present into the cell has been estimated to be ncRNA, while only 1% of RNA is messenger RNA intended to be translated into proteins. Some ncRNAs are housekeeping RNA, such as ribosomal or transfer RNA, which are present in a high percentage and have structural and well-characterized functions. Among a vast number of different ncRNAs, microRNA (miRNA) are a heterogeneous group of molecules, typically ranging from 19 to 24 nucleotides, present in a very low percentage, generally well below 1% and with a not completely clarified regulatory role (Djebali et al., 2012). At nuclear level, miRNA genes are translated by RNA Pol II into a relatively long primary miRNA transcript, known as pri-miRNAs, which assume a double-strand hairpin structure. In the nucleus, the microprocessor complex, formed by Drosha and DiGeorge syndrome critical region 8 (DGCR8) cleaves pri-miRNAs with removal of the 5′-capped and 3′-polyadenilated parts (Di Leva et al., 2014; Slack and Chinnaiyan, 2019). This intermediate shorter form, called pre-miRNA and of roughly 60 nucleotides in length, is then transferred to the cytoplasm *via* an Exportin 5 and Ran-GTP complex (Clancy et al., 2019). Here, pre-miRNA is further cleaved by the RNase III Dicer, which removes the transcript extremities and the hairpin loop originating the mature double-strand duplex, formed by two antiparallel filaments of roughly 22 nucleotides, the guide miRNA strand and its complementary miRNA. The guide miRNA strand then joints to an argonaut protein complex termed RNA-induced silencing complex (RISC), which allows a quite nonspecific interaction between a 3′ untranslated region (3′UTR) of target messenger RNAs and the few nucleotides nearby the 5′ end of the guide miRNA strand, known as the “seed”. The miRNA-mRNA interaction slows down mRNA translation and prompts for a faster mRNA degradation; in addition, the low specificity of this interaction increases consistently the number of target mRNAs and, consequentially, of the cellular activities affected by a single miRNA (Esquela-Kerscher and Slack, 2006). A schematic representation of miRNA intracellular synthesis is proposed in Figure 1.

### 1.2 miRNA and tumors

It should be considered that several hundred miRNAs exist (Kozomara et al., 2019; Alles et al., 2019): each one can interact with a wide number of cellular pathways but also can modulate the action of a vast number of other ncRNAs. These two interacting aspects of miRNA activity let us understand the outstanding importance of miRNAs in cancer pathogenesis. In fact, on the one hand miRNA and other ncRNAs create a complicated network of reciprocal influences, where all the components regulate each other. When in equilibrium, this network can impress its control on the synthesis of the almost totality of the cellular proteins, contributing to shaping the normal features of cell activities. On the other hand, the pathological perturbation of one or more elements often leads to a deep unbalancing of this network, which in turn can determine some strongly dysregulated patterns that can sustain cellular overgrowth, spreading and metastasis. Accordingly, miRNA networks have been found to be reprogrammed in all cancer types studied (Volinia et al., 2006; Volinia et al., 2010) exerting a strong influence on almost all the molecular and cellular pathways, including activation of critical oncogenes and tumor suppressors inhibition. In addition, when released in the extracellular environment, miRNAs are extremely stable, compared to other RNAs, also in the presence of high levels of RNAses. It is so not surprising that they have been detected into several body fluids, including ocular fluids, and have been proposed as biomarkers with diagnostic and prognostic value for many diseases, including cancer (Conti et al., 2020; Conti et al., 2021). In this scenario, we focus this review on the role of miRNA on adult ocular tumors.

### 1.3 Ocular malignant tumors

Ophthalmic cancers may be localized within the eye, named intraocular cancers, on the ocular surface, named conjunctival and corneal cancers, and in structures nearby the eye, named eyelid and orbital cancers. They can be both primary tumors and metastasis of tumors localized in distant sites; this review will focus on primary ocular tumors for which significant literature data on the role of miRNAs are available. Even if the overall frequency of ocular malignant tumors is low with respect to other types of cancer, the most frequent type of ocular cancer in adult patients is intraocular uveal melanoma (UM). UM has an incidence of 5.1 cases *per* million *per* year, with high rate of metastasis and short long-term survival (Kaliki and Shields, 2017). Primary lymphoma of the eye is another type of intraocular cancer that accounts for less than 1% of all ocular cancers and mostly affects the elderly or people with fragile immune systems (Melli et al., 2022). Tumors of the retina are extremely rare in adults and include hemangioblastoma, vaso-proliferative tumors, nerve fiber tumors and glioma, while retinoblastoma typically affects children under the age of 5, being classified as pediatric cancer (Bechrakis and Kreusel, 2011; Nag and Khetan, 2024). Conjunctival cancers include lymphoma, melanoma and squamous cell carcinoma, the most common cancer of the conjunctiva (Shields and Shields, 2019). Squamous cell carcinoma usually affects elderlies and is a low-grade tumor, growing slowly and locally; on the contrary, aggressive squamous cell carcinoma is mostly associated with AIDS (Gichuhi and Irlam, 2007). Since conjunctiva is a membrane that covers both the cornea and the inside part of the eyelid, these tumors can be considered affecting the ocular surface or the eyelid, depending on their anatomical localization. Finally, cancers of the eye’s nearby structures can affect the upper or lower eyelid, the eyelid-associated soft tissues (such as rhabdomyosarcoma), the skin (such as melanoma, squamous cell carcinoma, and the most common basal cell carcinoma), the lacrimal gland, and the sebaceous gland (Mukarram and Khachemoune, 2024; Silverman and Shinder, 2017).

In the next sections we review the recent discoveries that demonstrate a role for miRNA in UM, tumors of the retina and eyelid cancers.

## 2 Role of miRNA in uveal melanoma

### 2.1 Uveal melanoma

**FIGURE 2:**
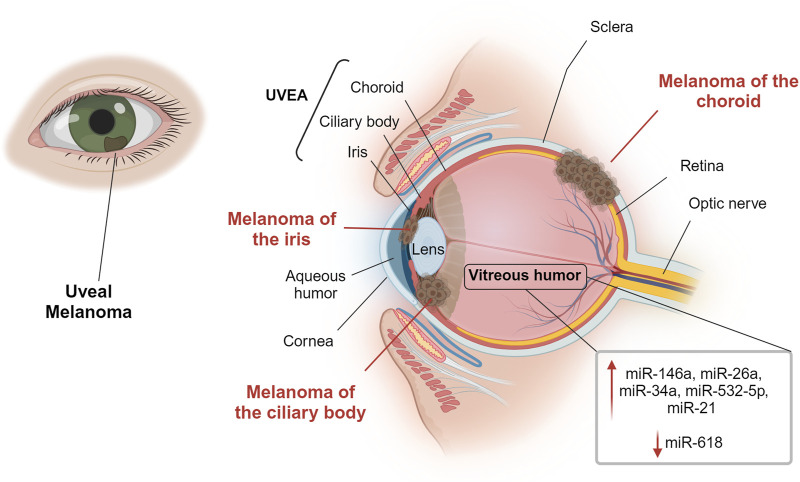
Overview of the eye and uveal melanoma sites. Uveal melanoma is the most common intraocular primary malignant tumor in adults. It can develop in the iris, in the ciliary body or in the vascular choroid of the posterior chamber of the eye. The figure indicates miRNAs localized in the vitreous humor that may have an important role in uveal melanoma. Created with BioRender.

**FIGURE 3:**
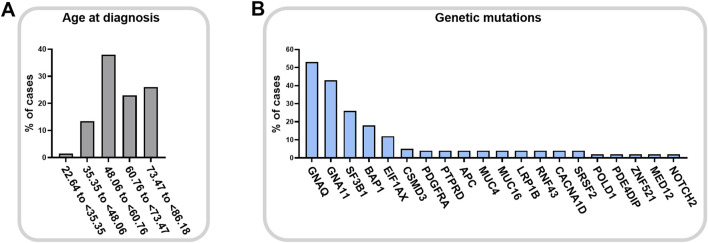
Uveal melanoma of the choroid onset. Age at diagnosis **(A)** for posterior uveal melanoma (cohort: EYE uveal melanoma, choroid melanoma, 67 cases) and relative gene mutation frequencies **(B)** (TCGA search 12 April 2024, NIH GDC Data Portal).

Melanoma is a malignant tumor showing a high mortality rate. In the eye, melanoma rarely affects the bulbar conjunctiva, having as preferred sites the uveal structure, e.g. iris, ciliary body (region behind the iris that produces the aqueous humor) and the vascular choroid in the posterior chamber of the eye (McLaughlin et al., 2005), as represented in Figure 2. This makes UM the most frequent form of ocular melanoma and accounts for roughly 5% of all melanomas (Krantz et al., 2017). Epidemiological data suggest that pigmentation more than light exposure can be associated with UM incidence (Virgili et al., 2007; Hu et al., 2005). Light/fair skin and eye color appear to be significantly associated with UM, but there is no concordance on the relevance of UV light as a risk factor (Shah et al., 2005). Mean age of diagnosis is 62 years, with a prevalence in the age group between 48 and 60 years old, Figure 3A, quite variable in different ethnic groups, while age-adjusted incidence of UM is roughly 30% higher in males in respect to females (Kaliki and Shields, 2017). Although this cancer is rare in the total population, the risk of developing metastases is unfortunately very high (in about 50% of cases), and, if not detected early, the risk of lethality remains significant because the tumor responds poorly to chemotherapy (Jager et al., 2020).

Oculodermal melanocytosis and the presence of intraocular nevi increase the risk of developing UM, together with mutations in the BRCA1 associated protein 1 (*BAP1*). *BAP1* is an onco-suppressor gene involved in DNA damage response, apoptosis, and chromatin remodeling, and its loss-of-function mutations have been found in a high percentage in UM (Harbour et al., 2010), and in other cancers, as skin melanoma, mesothelioma, renal cell carcinoma (Carbone et al., 2013). *BAP1* inactivation is frequently due to monosomy 3 and it is associated with high risk of metastases and poor prognosis (Farquhar et al., 2017; Pašalić et al., 2023; Vader et al., 2017). Moreover, also aberrations of chromosome 1, 6 and 8, and mutations in *SF3B1* gene concur to metastatic potential and to the worsening of survival rate (Pašalić et al., 2023; Vader et al., 2017). It is interesting to note that *TP53* gene, which is the most frequently mutated gene in tumors (mutated in about 30% of all tumors), is not altered in UM (NIH GDC Data Portal data, https://portal.gdc.cancer.gov/). It is therefore clear that the mutations in UM have a very specific fingerprint, represented in Figure 3B, with the most prevalent mutations affecting *GNAQ* and *GNA11* genes (Vader et al., 2017). Mutations in these genes lead to constitutive activation of the G protein-coupled receptor, driving tumor carcinogenesis by promoting specific intracellular pathways, including MAPK, PI3K/Akt, and YAP/TAZ pathways. *GNAQ* and *GNA11* mutations are almost exclusive of UM and arise in the early stages of UM. However, contrasting data regarding their implication on tumor progression and overall survival are reported in the literature (Silva-Rodríguez et al., 2022; García-Mulero et al., 2023). Initial observations in human tissues and mice reported an association of GNAQ and GNA11 mutations as major contributors to UM development. However, other studies reported no association with disease-free survival and progression, supporting the idea of a greater role in early events of carcinogenesis (Silva-Rodríguez et al., 2022). The different types of tissues studied, the different number of samples considered and the possible association with other interfering factors cannot still define the role of these mutations in UM progression, however, they agreed about their importance as a possible therapeutic target.

### 2.2 miRNA in UM primary tumor tissues

In 2008, a work by the group of Worley screened genome-wide miRNAs expression in 24 UM cryopreserved samples by microarray (Worley et al., 2008). They were able to cluster patients in low and high metastatic risk groups discriminating with levels of both **let-7b** and **miR-199a**. After this discovery, several studies have investigated the role of miRNA by analyzing primary UM tumors and evaluating their relevance in prognosis and metastasis. We briefly summarize here the most relevant results.

In 2016, two independent groups correlated miRNA alterations along with monosomy-3 in primary tumoral tissues, with different results (Triozzi et al., 2016; Venkatesan et al., 2016). Triozzi et al. profiled 55 cryopreserved tumors (33 with monosomy-3 and 22 without) by microarray, identifying 6 over- and 19 under-expressed miRNAs in monosomy, and further validated the upregulation of **miR-92b** by qRT-PCR in a sub-cohort of 20 patients (Triozzi et al., 2016). Venkatesan et al. firstly profiled by microarray 6 formalin-fixed paraffin-embedded (FFPE) UM samples, and afterwords they validated the common pool of significantly dysregulated miRNA by qRT-PCR in a larger cohort of 51 monosomy-3 and 35 disomy FFPE-UM samples and in 10 UM cryopreserved samples. They found that **miR-134, miR-143, miR-146b, miR-199a** and **miR-214** were significantly overexpressed in monosomy-3 samples. Moreover, they correlated higher expression of **miR-134** and **miR-149** with liver metastasis (Venkatesan et al., 2016). Later, overexpression of **miR-592** was associated by another research group with chromosome 3 monosomy analyzing FFPE primary tumors of a cohort of 46 patients affected by choroidal UM (Wroblewska JP, 2020). A differential expression of specific miRNAs in disomy-*versus* monosomy-3 was confirmed by Souri et al., which had previously described an association between chromosome 3/BAP1 loss and inflammatory phenotype in UM (Souri et al., 2021). In this study, Soury et al., suggested that BAP1 loss could worsen the prognosis through specific sets of inflammation regulating miRNAs. In particular, analysis of 64 UM cryopreserved samples showed a significant upregulation of **miR-22, miR-155**, and **miR-635** in tumors with monosomy-3 aberration. Another study on 26 cryopreserved tumors derived by enucleated eyes compared the expression of miRNAs in high-, intermediate- and low-risk UM patients, stratified based on clinical, histological and molecular characteristics (Smit et al., 2019). This work identified 9 significant downregulated and 5 upregulated miRNAs in the high-risk group as potentially involved in metastasis. The upregulated miRNAs included **miR-132**-5p, **miR-16**-5p and the oncomirs **miR-151a**-3p, **miR-17**-5p and **miR-21**-5p.

Concordantly with other data (Venkatesan et al., 2016; Wroblewska et al., 2020; Worley et al., 2008), **miR-592** and **miR-199a** were found to be the most altered miRNA in UM following bioinformatic analysis of the TCGA UM database (Falzone et al., 2019). Interestingly, after comparing the expression profile of sera from 14 UM patients and controls resulting in upregulation of **miR-146a,** validation was obtained in serum and in the FFPE-matched samples and 5 controls of choroidal melanocytes from unaffected eyes (Russo et al., 2016). Although limited by the small number of patients, this study certainly has the merit of having used healthy choroidal tissue as control, and of having shown that **miR-146** is significantly overexpressed in the tumor as well as in the serum of patients.

### 2.3 Extracellular UM-associated miRNA

In addition to cytoplasmic markers, circulating biological markers have been found to be very helpful in addressing the onset and recurrence of a tumor, in establishing the stage and in predicting the response to treatment. Since UM is a high-risk metastatic cancer, an efficient liquid biopsy investigating a specific panel of miRNAs could be significant in saving lives. In this section we revise the more relevant literature presenting significant miRNA dysregulation in serum/plasma and ocular fluids of UM patients.

#### 2.3.1 Serum miRNA

Some studies have reported specific miRNA profiles alterations in serum or plasma of UM patients with respect to controls.

Plasma levels of **miR-20a, miR-125b, miR-146a, miR-155, miR-181a,** and **miR-223** were initially found upregulated in 6 UM patients at diagnosis compared to 26 controls (Achberger et al., 2014), and **miR-146a** was independently confirmed upregulated in the serum of 6 UM patients recruited for miRNA profiling of both blood and vitreous humor (Ragusa et al., 2015). A more extensive analysis came from the transcriptome profiling of 754 miRNAs in serum of 14 UM patients and 14 healthy controls, resulting in upregulation in the patient’s samples of **miR-146a** and **miR-523** and downregulation of **miR-19a**, **miR-30d**, **miR-127**, **miR-451**, **miR-518f** and **miR-1274b**. Further data validation confirmed the upregulation of **miR-146a** in serum, as well as in tumoral tissues of these patients (Russo et al., 2016). Moreover, elevated plasma levels of **miR-92b, miR-199a-5p** and **miR-223**, were shown upregulated in 65 UM patients compared to normal 26 controls and were significantly higher in patients with monosomy-3 respect to patients without the chromosome-3 deletion, indicating a prognostic value for this miRNA triad (Triozzi et al., 2016). Furthermore, a multicenter cross-sectional study of 17 miRNAs panel on serum of 55 UM patients and 10 uveal nevi-affected controls measured by qRT-PCR, showed that six miRNAs were significantly upregulated in UM: **miR-16, miR-145, miR-146a, miR-204, miR-211** and **miR-636-3p** (Stark et al., 2019). More recently, sera of a cohort of 25 patients with choroid UM and 10 healthy donors were investigated to validate a small panel of circulating miRNA by qRT-PCR (Zhou et al., 2022). In this work, **miR-199a-3p** and **miR-21-5p** were identified as new promising circulating biomarkers for diagnosis and prognosis, respectively.

Some studies investigated the role of miRNAs in UM metastasis. **miR-21-5p** was found upregulated in serum of metastatic patients respect to non-metastatic patients and controls (Zhou et al., 2022), as well as levels of **miR-211** significantly distinguished localized and metastatic UM tumors (Stark et al., 2019). Interestingly, in a time-course clinical observations of 6 UM patients since their primary diagnosis, plasma levels of **miR-20a, miR-125b, miR-146a, miR-155, and miR-223** increased further respect to diagnosis, and **miR-181a** decreased, during metastasis progression (Achberger et al., 2014).

#### 2.3.2 Extracellular UM-associated miRNA in ocular fluids

Most of the publications on the presence of miRNAs in the ocular fluids of UM patients are the works of Marco Ragusa’s team that studied the miRNAs profile in the vitreous humor of patients who underwent vitrectomy or enucleation, and that suggested some clinical implications about 10 years ago (Ragusa et al., 2013; Ragusa et al., 2015; Barbagallo et al., 2021). In these pioneer works, the authors demonstrated for the first time that miRNAs were available in the vitreous humor and that UM samples overexpressed some miRNA respect to controls with a unique profile, suggesting that **miR-26a, miR-34a, miR-146a** and **miR-532-5p** can have a potential role in UM (Ragusa et al., 2013). Moreover, they observed that most miRNAs were included in vitreal exosomal nanovesicles, confirming increased expression of **miR-34a, miR-146a** and **miR-21** in both vitreous humor and vitreal exosomes samples from UM patients respect to unaffected ones (Ragusa et al., 2015). In the same work, **miR-618** instead was found to be downregulated in vitreous humor and upregulated in vitreal exosomes. The authors integrated the data about local miRNA secretion with analysis of serum circulating miRNA, observing again upregulation of **miR-146a** and **miR-618**. Lastly, they found that **miR-146a** and **miR-21** were also increased in UM tissues. Based on these observations, they indicated **miR-146a** as a potential circulating biomarker of UM (Ragusa et al., 2015). Nonetheless, the studies suffer from the small cohorts investigated (6 and 18 patients) and need to be expanded before speculating on their effective diagnostic use.

More recently, the group of Jesse L. Berry made a series of quantitative assessments in aqueous humor of two cohorts of UM patients (a first pilot of 20, and a second analysis of 66 patients) with the purpose to evaluate which classes of molecules were available and sufficient to perform successful analyses in the samples (Im et al., 2022; Pike et al., 2023). They recognized aqueous humor as a relevant source of miRNAs (as well as of dsDNA and proteins) whose concentration increases with tumor stage, tumor size, and after brachytherapy treatment. Data are not qualitative so far, but they indicate that the concentration of analytes depends on the tumor.

Most of the literature on UM and ocular fluids focuses on the study of protein biomarkers, but the studies reviewed here point to the possibility of future advances and more deepening on miRNAs analyses using these specimens. In Figure 2, miRNAs that could have an important role in UM are reported. It will be of great interest to understand similarities and differences between miRNAs in the anterior aqueous humor or in the posterior vitreous humor, and possible relationships with the tumor onset site (iris, ciliary body or choroid) and metastasis.

For clarity, those miRNAs that are reported in at least two different types of UM biological samples, tumor tissues, serum/plasma or ocular fluids, with results that were reproducible in different specimens and that can have a potential as biomarkers for this pathology, are summarized in Table 1.

**TABLE 1:** Selected miRNAs showing deregulated expression in different UM patients’ specimens.

miRNA	Biological sample	References	Evidence
miR-146	Tumor tissues	Russo et al., 2016 Venkatesan N (2016)	miR-146a overexpressed miR-146b overexpressed in tumors with monosomy-3 aberration
	Serum/plasma	Achberger et al., 2014 Ragusa et al., 2015 Russo et al., 2016 Stark et al. (2019)	miR-146a increased levels in plasma and serum and higher in metastatic patients
	Ocular fluid	Ragusa et al., 2013 Ragusa et al. (2015)	miR-146-a increased levels in vitreous humor and in vitreous exosomes
miR-21	Tumor issues	Smit et al. (2019)	miR21-5p overexpressed in metastasis high-risk patients
	Serum/plasma	Zhou et al. (2022)	miR21-5p upregulated in serum, particularly in metastatic patients
	Ocular fluid	Ragusa et al. (2015)	Increased levels in vitreous humor and in vitreous exosomes
miR-199	Tumor tissues	Worley et al., 2008 Venkatesan et al. (2016)	miR-199a overexpressed in tumors with monosomy-3 aberration and in patients with metastasis high-risk
	Serum/plasma	Triozzi et al., 2016 Zhou et al. (2022)	miR-199a-5p upregulated in plasma and higher in patients with monosomy-3 aberration; miR-199a-3p upregulated in serum of metastatic patients
	Ocular fluid	N/A	
miR-155	Tumor tissues	Souri et al. (2021)	Overexpressed in tumors with monosomy-3 aberration
	Serum/plasma	Achberger et al. (2014)	Upregulated in plasma and higher in metastatic patients
	Ocular fluid	N/A	
miR-16	Tumor	Smit et al. (2019)	miR-16-5p overexpressed in metastasis high-risk patients
	Serum/plasma	Stark et al. (2019)	Upregulated in serum
	Ocular fluid	N/A	
miR-92b	Tumor	Triozzi et al. (2016)	Overexpressed in tumors with monosomy-3 aberration
	Serum/plasma	Triozzi et al. (2016)	Upregulated in plasma; higher levels related with tumor monosomy-3 aberration
	Ocular fluid	N/A	

### 2.4 miRNA regulating p53 pathway in UM

**FIGURE 4:**
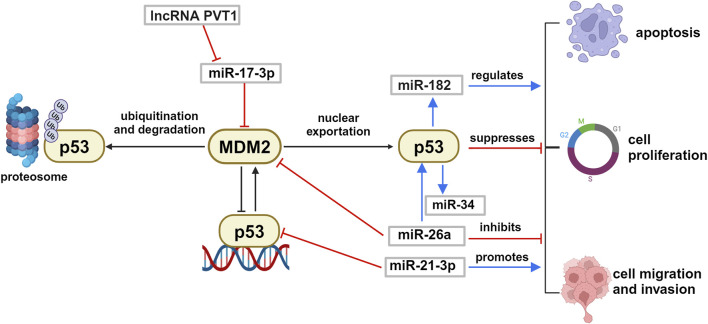
Schematic representation of p53/MDM2 axis regulation by ncRNA in uveal melanoma. p53 and MDM2 reciprocally control each other by a negative feed-back loop. The p53/MDM2 axis is regulated by several ncRNAs in uveal melanoma, that can interfere with the p53-driven biological effects such as cell proliferation, apoptosis, migration and invasion. Created with BioRender.

As stressed before, the genetic mutations in the *TP53* gene, expressing the protein p53 known as guardian of genome, are absent or rare in UM (cfr Figure 3B). Considering that p53 is fundamental for the cellular decision about survival and death, as well as for other biological events such as proliferation, migration and invasion, the identification of miRNAs able to regulate p53 expression level or p53 pathway can be relevant to control UM cell survival. For this reason, we revise here the relevant literature that demonstrated in UM anti-tumoral effects of specific miRNAs (and long non-coding RNA, lncRNA) that can activate p53 or target its inhibitor murine double minute 2 (MDM2) (Figure 4).

Wu and colleagues have shown that lncRNA PVT1 was overexpressed in UM tissues and that silencing of this non-coding RNA suppressed the proliferation, migration, and invasion of uveal melanoma cell lines *in vitro* (Wu et al., 2019). In a similar way, *in vivo* silencing of lncRNA PVT1 reduced the tumorigenic ability of uveal melanoma in nude mice. In addition, they showed that PVT1 oncogenic biological action was mediated by sequestering and inactivating **miR-17-3p**, which led in turn to upregulation of the protein MDM2, followed by the inactivation of the master oncosuppressor p53 protein. These data are supported by several studies that demonstrated the existence of a miR-17-3p/MDM2/p53 axis, in different types of cancer including melanoma (Kim and Shohet, 2009). The data strongly suggest that silencing of lncRNA PVT1 and overall overexpression of miR-17-3p could restore the well-known antitumoral activities of p53 and reduce *in vivo* UM tumorigenicity.

Further experimental *in vitro* confirmation of the importance of the regulation of MDM2/p53 axis came from the work of Guo who stated that **miR-26a** can act as a tumor suppressor miRNA and regulate this signaling pathway decreasing MDM2 and increasing p53 expression levels, which resulted in reduced viability of UM cells (Guo and Tian, 2020). Another miRNA affecting the pathway is the oncomiR **miR-21-**3p that targets directly *TP53* expression. Wang demonstrated *in vitro* and *in vivo* models of UM that silencing of miR-21-3p inhibited UM proliferation and migration, significantly reducing tumor growth in mice (Wang et al., 2018). Among miRNA transcriptionally controlled by p53, with relevance for ocular diseases, there are miR-182 and miR-34 families. It has been demonstrated that **miR-182** transiently transfected in UM cells reduced growth and invasiveness of the cultures, functioning as a tumor suppressor (Yan et al., 2012).

Interestingly, it has been observed in other cancer models that overexpression of miR-182 can have opposite effects, stimulating migration and invasion (Madrigal et al., 2023). It appears evident that these antithetic roles of miR-182 are finely controlled by the complex p53-dependent regulation of cellular life and it suggests specific modulation in cells of different origin. Another transcriptional target of p53 is the **miR-34** family that has been indicated as a critical mediator of p53 function and tumor suppressor (Navarro and Lieberman, 2015). miR-34 dysregulation is associated with several types of cancers, including retinoblastoma (Pan et al., 2023; Zauli et al., 2011; Dalgard et al., 2009). Interestingly, increase of miR-34a expression has been associated with retina and RPE aging, together with DNA damage in mitochondria, suggesting its involvement in senescence and apoptosis during ocular aging process (Smit-McBride et al., 2014), and in age-associated inflammation (Badi et al., 2015). These events could favor a microenvironment that can predispose eye tumoral development in adults and elderly.

Considering these experimental observations, it emerges that the modulation of the miRNAs involved in the p53 pathway could be a good therapeutic strategy for UM.

## 3 Role of miRNA in the adult tumors of the retina

### 3.1 Tumors of the retina

**FIGURE 5:**
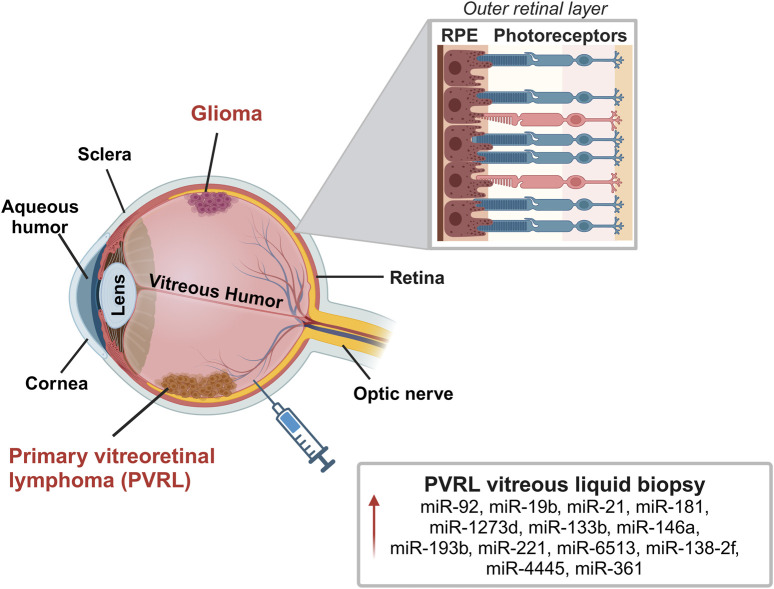
Representation of the most common retinal tumors in adults: intraocular lymphoma and gliomas. Primary vitreoretinal lymphoma (PVRL) is a rare type of central nervous system lymphoma (PCNSL) that affects the retina, vitreous and in some cases also the optic nerve. Glioma is a rare central nervous system tumor that affects the retina and optic nerve. The image reports miRNAs localized in the vitreous humor that may play a role in PVRL. In the zoomed panel it is reported a schematic structure of the outer layer of the retina with interaction between photoreceptors and retinal pigmented epithelial (RPE) cells. Created with BioRender.

The retina covers the posterior part of the eye. It consists of several cell layers: photoreceptors (cones and rods), retinal ganglion cells, bipolar cells, horizontal cells, amacrine cells, Müller cells and retinal pigment epithelium cells (RPE). The innermost layers, representing the neurosensory retina are adjacent to the vitreous humor, while the outermost layer is the RPE (Casciano et al., 2024). RPE helps in light collection and participates in the formation of the blood-retina barrier avoiding direct diffusion of nutrients from the vascular choroid (Casciano et al., 2024; Hurley, 2021). Several miRNAs control retinal homeostasis and functionality, and loss of their regulation has been reported in retinal disorders (Zuzic et al., 2019). Tumors of the retina can develop from different types of cells, including retinal cells (photoreceptors and glial cells), vascular endothelium, RPE, or rarely also from tumor metastasis, mainly from breast cancer, lung tumor, and cutaneous melanoma (Bechrakis and Kreusel, 2011). In adults, the most common cancers affecting the retina are intraocular lymphomas and gliomas (Figure 5).

Primary vitreoretinal lymphoma (PVRL) is a rare type of central nervous system lymphoma that affects the retina, the vitreous and in some cases also the optic nerve. PVRL is the most frequent type of diffuse-B cell lymphoma of the eye typically arising in the fifth/sixth decade of life as malignant bilateral tumor (Kalogeropoulos et al., 2019; Sobolewska et al., 2021).

Retinal functions and homeostasis are assured by retinal glial cells, primarily represented by Müller cells followed by astroglia and microglia. Glia physiologically supports and provides nutrients to retinal neurons, but they can also promote the release of pro-inflammatory mediators (Jiang et al., 2024; Kobat and Turgut, 2020). Gliomas affecting the retina and optic nerve are rare central nervous system tumors that, in many cases, are reported in children as benign neoplasms, while in adults they represent malignant tumors. Patients rapidly lose their vision unilaterally and then bilaterally with only a few months of life expectancy (Dal Bello et al., 2023; Ostrom et al., 2014). Increased proliferation of glial cells and astrocytes has been evidenced as an initial sign of tumor onset, promoting tumor growth by cytokines, chemokines, and growing factors release (Dal Bello et al., 2023).

Diagnosis of cancer affecting the retina can be challenging with delays in treatment with increased morbidity and mortality (Dawson et al., 2018; Sobolewska et al., 2021). PVRL, for instance, is often confused with uveitis due to unsuitable diagnostic tools such as ophthalmoscope. The chorioretinal biopsy provides a more appropriate diagnosis, however, alternative methods would be preferred to reduce the invasiveness. In this context, some research analyzed potential molecules, including miRNA secreted into vitreous humor, as biomarkers for differential diagnosis also for these types of tumors (Minezaki et al., 2020; Tuo et al., 2014).

### 3.2 miRNA in tumors of the retina

The study by Tuo and colleagues on vitreous humor samples from 3 PVRL and 3 uveitis, first analyzed a panel of 168 miRNA reporting a significant upregulation only of **miR-155** in samples from uveitis vitreous compared to PVRL (Tuo et al., 2014). Kakkassery and colleagues reported upregulation of **miR-92**, **miR-19b** and **miR-21** in surnatant of vitreous biopsies from 10 PVRL patients with respect to patients with macular pucker. **miR-92** and **miR-21** expression was also upregulated in surnatant of PVRL compared to vitritis patients (Kakkassery et al., 2017). In agreement, **miR-21** upregulation was also reported by Minezaki and colleagues in vitreous humor of PVRL compared to patients with macular hole or epiretinal membrane (Minezaki et al., 2020). They first identified a specific miRNA profile expression in the vitreous and serum of vitreoretinal lymphoma patients that differed from controls. Over 2565 miRNA tested, they reported the upregulation in serum and vitreous of PVRL compared to control of **miR-1273d**, **miR-133b**, **miR-146a, miR-181**, **miR-193b**, **miR-221**, **miR-326**, **miR-345**, **miR-442**, **miR-4655**. When compared to uveitis, PVRL samples presented upregulation of **miR-6513**, **miR-138**-**2f** and **miR-4445**. The authors then suggested considering **miR-361** as a diagnostic marker to discriminate vitritis from uveitis among all tested miRNA. **miR-361** is already known to be modulated in solid tumors like colon, lung and retinoblastoma and the increased levels in vitreous humor of PVRL might support carcinogenesis (Melli et al., 2022; Minezaki et al., 2020). Figure 5 displays the most relevant miRNAs localized in vitreous humor of these patients.

Many miRNAs are involved in retinal glial cell regulation (e.g., proliferation, inflammation), generating a tumor-favourable environment; however, no specific studies reported miRNAs implication in the glioma of the retina (Caruso et al., 2024; Jiang et al., 2024). Nevertheless, conditions like hypoxia, cytokines and growth factors release were reported to enhance the expression of **miR-21** in glioma tissues and glioma cell lines, promoting tumor growth and invasion. Several *in vitro* and *ex-vivo* studies consistently reported **miR-21** upregulation in glioma, while its inhibition was associated with reduced proliferation and migration *in vitro* (Aloizou et al., 2020; Nieland et al., 2022). Similarly, increased expression of **miR-9** was reported to promote proliferation, migration, invasion and angiogenesis of glioma cells both *in vitro* and *in vivo* by degrading mRNA of protein functionally involved in HIF-1/VEGF pathway (Chen et al., 2019).

## 4 Role of miRNA in eyelid cancer

The most common form of eyelid cancer is basal cell carcinoma. Other types of eyelid tumors include malignant melanoma, sebaceous cell carcinoma, squamous cell carcinoma and lymphomas (Silverman and Shinder, 2017). Basal cell carcinoma (BCC) is the most common cancer in the world and 20% of these tumors affect the skin of eyelid. However, only a few studies reported the analysis of miRNA in BCC and to the best of our knowledge, no specific studies explicitly referred to eye-BCC. Studies on miRNAs dysregulation in the other eyelid cancer types are reported in the following subsections.

### 4.1 miRNA in sebaceous carcinoma

Sebaceous gland carcinomas (SGC) mainly occur in the upper lid and easily spread to periocular tissues (epithelium of the palpebra, bulbar conjunctiva and cornea) (Silverman and Shinder, 2017). It is a malignant tumor more common in the Asian population compared to the Caucasian with a high rate of metastasis and mortality. Hirano and colleagues identified 16 upregulated and 29 downregulated miRNAs in SGC compared to sebaceous adenoma using a small RNA-sequencing analysis. They also analyzed pathways in which these miRNAs might be involved, revealing a role in decreasing lipid metabolism and increasing cell survival and proliferation. Upregulation of **miR-130a** and **miR-939** was reported as crucial nodes in decreasing lipid metabolism, while downregulation of **miR-146a, miR-149, miR-193a, miR-195** and **miR-4671** was proposed as enhancer of cell proliferation (Hirano et al., 2021). Tetzlaff and colleagues compared SGC with sebaceous adenoma and reported **miR-195** and **miR-211** downregulation and **miR-486** and **miR-184** overexpression, suggesting a dysregulation of NF-kB and TGF-β signaling mediated by **miR-486** and **miR-184**, respectively (Tetzlaff et al., 2015). Bladen and colleagues conducted a sequencing study on different subtypes of SGC reporting **mirR-16** overexpression and **miR-34** downregulation as common dysregulated miRNA in all SGC subtypes analysed compared to tissues from healthy subjects (Bladen et al., 2018). Bhardwaj and colleagues compared miRNA expression in SGC tissues with healthy tissues from the same patient showing downregulation of **miR-200** and **miR-141** that promote epithelial-mesenchymal transition and correlate with large tumor size and shorter disease-free survival (Bhardwaj et al., 2017). Increased proliferation and migration were also reported in association with increased levels of **miR-3907** in primary cells derived from SGC tissue by Zhang and colleagues Zhang et al. (2021). An important promotor of epithelial-mesenchymal transition is represented by zinc finger E-box binding homebox 2 (ZEB2), a target gene of **miR-651** that has been reported to be downregulated in SGC tissues compared to adjacent normal tissue (Zhao et al., 2022).

### 4.2 miRNA and conjunctival tumors

Conjunctival melanoma (CM) is a rare cancer originating from melanocytes of the basal layer of the conjunctival membrane with frequent metastasis and a high mortality rate (Rossi et al., 2019). Studies focusing on miRNA deregulation in CM suggested potential prognostic or therapeutic targets (Larsen et al., 2016; Rossi et al., 2019). miRNA expression analysis on CM tissues compared with normal conjunctiva revealed downregulation of **miR-5096** and upregulation of **miR-132, miE-138, miR-363, miR-146a/b, miR-509, miR181, miR-500, miR-20b, miR-506, miR-532, miR-501,** all miRNA already identified also in cutaneous melanoma and associated with tumor progression and metastatic process (Larsen et al., 2016; Mikkelsen et al., 2019). Metastatic and non-metastatic CM has been reported to possess a separate pattern of dysregulated miRNA. Indeed **miR-4528, miR-1270, miR-1290, miR-548f-4, miR-4278 and miR-34a** were reported to be downregulated in primary CM vs. metastatic tissues, while **miR**-**575, miR-527, miR-518a, miR-6759, miR-8078, miR-4501, miR-622, miR-4698, and miR-4654** were downregulated (Mikkelsen et al., 2019). **miR-622** acts as a tumor suppressor in cutaneous melanoma and its downregulation together with the downregulation of **miR-1208** has been suggested to be mediated by increased levels of the circulating RNA MTUS1 (**circMTUS1**) reported in CM tissues compared to normal tissues (Shang et al., 2019).

### 4.3 miRNA and eyelid lymphoma

Lymphomas affecting the orbit and eyelid present several subtypes with different grades of invasiveness. Half of the tumors are extranodal marginal zone B-cell lymphomas, an indolent lymphoma also known as mucosa-associated lymphoid tissue (MALT) lymphomas (Savino et al., 2020). Cai and colleagues reported a significant upregulation of **miR-150** and downregulation of **miR-184, miR-200a, miR-200b, miR-200c, and miR-205** in conjunctival MALT compared with adjacent matched normal tissue. They further demonstrated that **miR-200** family overexpression in a human B cell line downregulates cyclin E2. Based on that they suggest that downregulation of miR-200 family *via* Cyclin E2 overexpression promotes the progression of lymphoma, as already reported for other cancers like breast, ovarian, gastric and pancreatic cancers (Cai et al., 2012). Moreover, results from the same research group reported that transfection of **miR-184** or **miR-205** were associated with apoptosis and reduced survival, migration and invasion (Li et al., 2022). Among the rare tumors affecting the ocular adnexal region, diffuse large B-cell lymphoma is among the most aggressive. It can be a primary cancer or, in some cases, derived from a MALT progression and transformation. A study comparing miRNA expression in this tumor with MALT tissues reported **miR-24** family, **miR-221/222**, **miR-23a** and **miR-29** cluster downregulation in addition to already known MYC suppressors like **miR-26a**, **let-7g** and **miR-221** (Hother et al., 2013).

## 5 Clinical implications of ocular cancers’ miRNA research: limitations, potentialities and applications

### 5.1 Clinical issues related to patient samples

**FIGURE 6:**
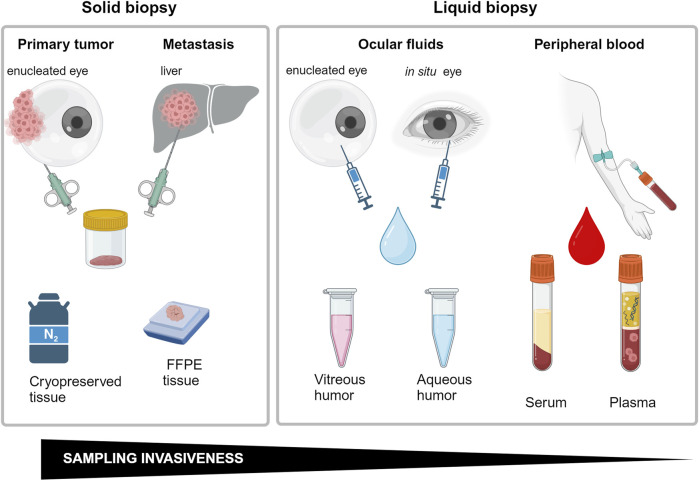
Schematic representations of biopsy methods employed to miRNA analysis correlated with invasiveness. Created with BioRender.

Transformation of a normal nevo into a primary UM, or *de novo* appearance of a UM lesion, are critical phases in the appearance of ocular melanoma that could not be recognized during routinary ophthalmoscopy. On the other hand, to perform a correct diagnosis in the very initial times of the illness, it is crucial to orient the therapeutical intervention choosing between a more conservative or a highly demolitive approach, such as eye enucleation. In addition, the size of the tumor at discovery is critical to determine the risk of developing metastasis and consequentially strongly influence the overall survival. In this anatomical district, the correct sampling is quite demanding and it can heavily influence the diagnostic and prognostic information obtainable by molecular assays performed to investigate the tumor biomarkers. It is possible to obtain biological material directly from the tumor performing quite invasive procedures (Figure 6). Fine-needle aspiration biopsy, for example, allows the collection of material from melanomas located in the anterior chamber, as iris tumors. Similarly, fine-needle aspiration can be used to reach the ciliary body and the parts of uvea and retina located anteriorly to the equator, with a trans-scleral approach. For the same locations, a more invasive excisional approach, with formation of a scleral flap, can be used. When tumor localization occurs in the posterior parts of the eye wall, access from *pars plana* with crossing of retina and vitreous body can be mandatory, both for fine-needle aspiration or more challenging vitrectomy surgery (Yang et al., 2021). With the same approaches, biologic material could be obtained from anterior chamber or from vitreous humor. The collected material could be used to obtain a miRNA expression profile to determine several other biomarkers, such as tumor cells, exosomes, circulating DNA, mRNAs and miRNAs.

To collect material for histological or other laboratory analysis directly from the tumor or its proximity is quite demanding. All these approaches are not free from some concerns, including the possibility of contributing to tumor spreading and the risk of worsening the patient’s sight, favoring complications including bacterial infections, hemorrhage, and retinal detachment. In addition, collection of insufficient amounts of samples for laboratory and histological analysis can sometimes not be decisive (Bande Rodríguez et al., 2020). For these reasons, these procedures are not frequently applied, and when preserving the affected eye is not possible, clinicians prefer to enucleate the eye to safeguard the patients’ life (Roelofsen et al., 2021).

### 5.2 Potentialities and limitations of liquid biopsy

For several cancers, liquid biopsy seems to represent a valuable and versatile add-on in the toolbox of the oncology specialist, that can overcome most of the problems joined to direct tumor sampling at ocular level. Blood can represent a valuable source of several different tumor-related biomarkers as circulating tumor cells, extracellular vesicles, cell-free DNA or RNA, miRNA, mRNA and proteins. Uvea is the vascular layer of the eye wall, and vessels can represent, without a well-defined ocular lymphatic system, the main route for disseminating all the material released by UM, including circulating tumor cells, exosomes, miRNAs and other circulating nucleic acids. Among all biomarkers, miRNAs have attracted increasing attention in force of some interesting features. From a technical point of view, circulating miRNAs (cmiRNAs) represent a class of RNAs that shows an excellent resistance to RNase, freeze-thaw cycles and pH changes. Interestingly, these important features are not restricted to miRNAs encapsulated into extracellular vesicles or complexed to circulating proteins, but also to the free circulating forms. This makes cmiRNAs suitable candidates to be tested as biomarkers to distinguish between presence or absence of tumor, responsiveness to therapy and tumor prognosis. However, it is necessary to always be aware that the quantity and quality of miRNAs circulating in plasma and serum do not exactly mirror the alterations of the specific tumor tissue but are a representation of how the whole body reacts to the homeostasis alteration due to the tumor. For this reason, it is difficult to attribute a direct role of a serum miRNA on the tumor itself, as well as to establish the tissue of origin of each specific miRNA (Barbagallo et al., 2021).

Moreover, even when a significant number of publications is available, such as in the case of UM, to design a matrix to cross-reference data of the various studies on circulating miRNAs is challenging. Usually, small cohorts of patients are involved in each different work and methods of purification and analysis are quite different. Indeed, some negative aspects of the purification methods need to be considered, e.g. the signal interference in serum due to cell breakage during blood clotting, and due to platelets presence in plasma. The differences in sample extraction and data analysis used by research teams worldwide are relevant and standardization is still missing, as discussed in the recent perspectives of Beasley et al. (Beasley et al., 2022). In the case of UM, the aim to identify a unique panel of miRNAs from UM tissues analysis is so far still unreached, since each cohort seems to have aberrations of different miRNAs (as discussed in the previous sections). Moreover, analyses on healthy melanocytes of the eye are rare. It would be interesting to analyze the tissues of the ocular nevi. This is clearly limited by the fact that eye biopsies have a high risk and an organ that has undergone enucleation for other ocular pathologies may not be suitable for several reasons. Cadaver donations, that is routinary for cornea transplantation, could instead be a significant source of healthy tissues (ocular nevi or purified ocular choroidal melanocytes), but, to our knowledge, data from this type of samples are not available so far.

### 5.3 Clinical trials

Nonetheless, the new discoveries in this field will open to innovative low-invasive strategies against ocular tumors, from diagnosis to prognosis and therapy. As far as we know, a multicentric clinical study is currently open and published on ClinicalTrials.gov. The study aims to evaluate deregulated miRNAs and their role in diagnosis and prognosis of UM in 51 patients with uveal melanoma and 51 age- and sex-matched controls (“The Role of Genetic Mutations and of Circulating mRNAs in Uveal Melanoma”, NCT05179174, www.clinicaltrials.gov). The blood sample of each patient enrolled in the trial is assessed by droplet digital PCR for *GNA11* and *GNAQ* mutations and for **miR-506-514 cluster**, **miR-592** and **miR-199a- 5p**. In the European Union clinical trials register 58 trials on uveal melanoma are displayed, but none with the explicit aim of miRNA evaluation (www.clinicaltrialsregister.eu). Similarly, in the Australian New Zealand clinical trials registry 65 trials evaluating UM are on-going without apparent miRNA analysis (www.anzctr.org.au). Interestingly, the Chinese Clinical Trial registry presents an observational study (registration time 2024/04/09) for the construction of a uveal melanoma biobank for several samples: tumor, paracancerous, vitreous, aqueous humor, tear, plasma and blood cells, that can serve as source for miRNA studies (Construction of Uveal Melanoma Registration Platform and Sample Bank; registration number: ChiCTR2400082833). Even if no other clinical trials appear to be evaluating miRNAs in ocular cancers, several settled and certified research biobanks fulfilling standard guidelines are biobanking ocular tumors. Examples are the Nice Ocular MAlignancy (NOMA) Biobank of Nice, France, (Martel et al., 2023), the Ocular Oncology Biobank (OOB) of Liverpool, UK, the Xiangya Ocular Tumor Bank of Changsha, China (Gao et al., 2022). These biobanks are fundamental sources of samples and clinical data that will favor the necessary deepening in ocular cancers research, and in miRNA role evaluation, helping in advancing clinical translation for these diseases.

## 6 miRNA nanomedicine for the eye

Since UM are characterized by a small number of genetic lesions, the restoring of the dysregulated epigenetic control can assume relevance (Yang et al., 2021). A generally accepted strategy consists of providing underexpressed tumor suppressor miRNAs or counteracting overexpressed oncoMirs by furnishing antiMirs (Bader et al., 2010) to sequester dangerous upregulated miRNAs. These strategies appear very promising because they allow to influence a wide range of intracellular pathways, but in turn they can depend on the delivery route chosen to target the tumor *in vivo*. Direct intravenous administration or periocular injections can be challenging (Mack, 2010; Ranta et al., 2010), in force of the presence of RNAses, dilution factors and different types of anatomical barriers, ultimately leading to low intracellular loading at the tumor site. Intravitreal injection suffers from several constraints, as discussed above, and several injections may be required to warrant an adequate amount of drug. Interestingly, Wan et al. proposed the suprachoroidal space, the virtual space between sclera and choroid, as a suitable site to inject high concentration of drugs that will be slowly released in the posterior part of the eye (Wan et al., 2021). Consistently, Shen et al. observed that nanoparticle-mediated gene transfer *via* suprachoroidal route warranted long gene expression times in a safe and repeatable manner (Shen et al., 2020).

In addition, it could be strongly advisable to provide therapeutic miRNAs inside a protective core to protect miRNAs and optimize their transfer, minimizing degradation, reducing cargo leak, and maximizing uptake after specific release. Several strategies have been explored, including viral vectors and nanoparticle delivery (Shi et al., 2017; Yang et al., 2021). Viral vectors show high transfection efficiency which is counterbalanced by immunogenicity and intrinsic oncogenic potential. An interesting alternative is represented by nanoparticles (NP), that are characterized by low immunogenicity and high safeness degree. Moreover, they can be engineered with several internal and surface modifiers, useful to control a wide range of specific molecular interactions. NP can be divided into inorganic NP, based on carbon, silica and different metals, including iron and gold, and organic NP, characterized by several types of polymers or lipids, these ones arranged to form micelles or liposomes (Yang et al., 2021), Figure 7. Regarding NP approaches on ocular tumors, we review here the recent literature regarding their use as miRNA delivery systems.

**FIGURE 7:**
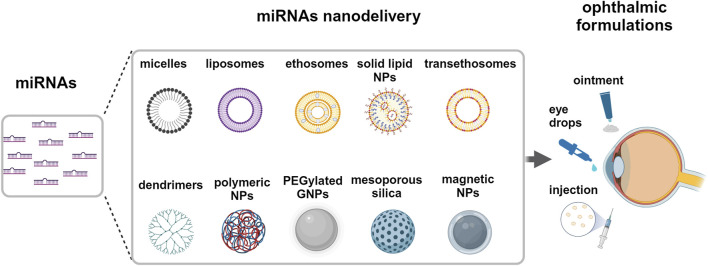
Delivery systems for miRNA to the eye. Potential strategies of miRNAs nanodelivery by different nanoparticles *via* topical, intraocular and periocular ophthalmic formulations. Created with BioRender.

Ma and colleagues used hybrid NP, redox-responsive *quasi*-mesoporous magnetic nanospheres, which exhibited a shell of polyethyleneimine stabilized by disulfide bonds, and an internal iron oxide core (Ma et al., 2023). They loaded the nanospheres with **miR-30a-5p** with high capacity in force of the strong electrostatic miRNA interaction with the nanosphere core. NP were successfully reduced and degraded by intraocular or intracellular glutathione, warranting a high release of miR-30a-5p. Typically, nanospheres were uptaken by endocytosis into UM cell lines in a dose-dependent manner and localized preferentially at perinuclear space. The uptaken miRNA significantly reduced UM cell migration and viability *in vitro*, targeting the E2F family of transcription factors and inducing apoptosis. The authors also performed *in vivo* experiments and reported that in a nude-mice model of UM, the tumor volume and weight were significantly reduced after the intraocular injection of miR-30a-5p-loaded nanospheres. In addition, they suggested that the nanosphere core, phagocytized by local macrophages, stimulated the macrophage shift toward an M1-like antitumoral phenotype, promoting macrophage activation and immunity response against the tumor. They concluded that redox-responsive nanospheres are suitable carriers for efficient miRNA delivery that can highlight antitumor miRNA activities *in vivo* in UM.

The use of miRNA-loaded exosomes has been found to be of therapeutic interest in the treatment of ailments affecting the external ocular surface, particularly the corneal interface. They seem to be a promising therapeutic approach in treating corneal neovascularization, being simply delivered at ocular level as eye drops (Twari et al., 2021; Mukwaya et al., 2019). Over the past decade, liposomal formulations have been significantly explored in ophthalmic drug delivery applications due to their ability to encapsulate both the hydrophilic and hydrophobic drug molecules and the ability to penetrate the corneal surface (Mishra et al., 2011). miRNAs can be easily loaded in liposomes by their chemical interactions between the positive charge of the nanocarrier and the negative charge of the transcripts (Urbán-Morlán et al., 2010). For instance, it has been demonstrated that **miR-34a** liposome is an effective therapy in orthotopic mouse models of neuroblastoma and liver cancer (Di Paolo et al., 2020; Daige et al., 2014). Liposomes, ethosomes, and trans-ethosomes could be potential nanocarriers for delivering miRNAs in uveal melanoma and other eye cancers (Yang et al., 2021). Li and colleagues have reported a safe *in-vivo* delivery of drug-loaded liposomes to the posterior segment of the eye by intravitreal injection (Li et al., 2022). Esposito and colleagues demonstrated that liposomes carrying nutlin-3a, a small molecule MDM2-inhibitor, can be potential candidates for vitreoretinal diseases (Esposito et al., 2024b). To reduce intraocular pressure, Uner and colleagues proposed timolol-loaded ethosomes for ophthalmic delivery (Uner et al., 2023). Trans-ethosomes have recently been formulated as gel with ketoconazole, an antifungal molecule, and showed to penetrate the cornea and to reach the posterior eye without toxicity (Ahmed et al., 2021). Similarly, trans-ethosomes have been used for drug delivery of low hydrophobic molecules able to activate p53 pathway, tested on skin epithelium and suggested as ophthalmic formulations (Esposito et al., 2024a; Romani et al., 2022; Casciano et al., 2023).

## 7 Conclusion

miRNAs have attracted great attention by scientists, in force of the pivotal role they play in the modulation of the almost totality of cellular functions and intercellular communication, both in normal and even more in tumoral cells. In fact, not only bioinformatic approaches have shown in tumors a deep alteration of the miRNA network, but also functional analyses have demonstrated *in vitro* and *in vivo* a strong regulative role on the activity of several proteins and axes, including ERK, MAPK, p53, cyclins and others. This makes miRNA excellent candidates as diagnostic/prognostic factors, and as targets for therapeutic interventions. This can be of outstanding importance for ocular tumors, and UM in particular. In fact, medical intervention at ocular level for sample collection is now the preferred choice but requires specialized surgery. The finding of diagnostic or prognostic factors directly obtainable from the blood could be a real improvement for patient safety and management. On this path, researchers are encouraged by miRNA presence and stability in the blood, both in the free form or encapsulated into lipidic exosomes. Several concerns exist and hinder the achievement of this important goal: circulating miRNA do not seem to mirror the dysregulation of primitive tumoral tissue, but are in general influenced by body health conditions, including, not last, the mere physical activity. If this finding seems to restrict the possibility to obtain diagnostic miRNA to direct ocular sampling, it also hampers but does not preclude in principle the possibility to obtain prognostic miRNA from blood. This should be of outstanding importance to forecast and manage the presence of metastases, which represent the principal reason of the ocular tumor’s lethality. Anyway, the number of miRNAs assumed to be important to regulate tumor biology and to signal cancer progression is quite vast, also in force of the relatively small number of patients enrolled in the different scientific studies, and the differences in sampling and analysis procedures, which gives a high degree of variability in the results observed. Another source of variability can come from the use of cell lines, which, although specific, can contribute with genetic and chromosomal aberrations joined to *in vitro* culture. Similar considerations can be drawn for identifying miRNA as potential pharmacological targets, to be silenced or overexpressed and results of clinical trials are, to date, few and variable.

To conclude, further studies with greater number of cases and standardized analysis approaches are required to obtain further insight on the role of miRNA in ocular tumor biology, to better manage with new therapeutic options diseases so disabling and very often also lethal.
